# Patient Perceptions and Potential Utility of Pharmacogenetic Testing in Chronic Pain Management and Opioid Use Disorder in the Camden Opioid Research Initiative

**DOI:** 10.3390/pharmaceutics14091863

**Published:** 2022-09-03

**Authors:** Dara Kusic, Jessica Heil, Stefan Zajic, Andrew Brangan, Oluseun Dairo, Gretchen Smith, Diego Morales-Scheihing, Russell J. Buono, Thomas N. Ferraro, Rachel Haroz, Matthew Salzman, Kaitlan Baston, Elliot Bodofsky, Michael Sabia, Alissa Resch, Laura B. Scheinfeldt

**Affiliations:** 1 Coriell Institute for Medical Research, Camden, NJ 08103, USA; 2 Cooper University Health Care, Camden, NJ 08103, USA; 3 GSK, Collegeville, PA 19426, USA; 4 Geisinger, Danville, PA 17822, USA; 5 Cooper Medical School of Rowan University, Camden, NJ 08103, USA

**Keywords:** pharmacogenetic, opioid, chronic pain, OUD

## Abstract

Pharmacogenetics (PGx) has the potential to improve opioid medication management. Here, we present patient perception data, pharmacogenetic data and medication management trends in patients with chronic pain (arm 1) and opioid use disorder (arm 2) treated at Cooper University Health Care in Camden City, NJ. Our results demonstrate that the majority of patients in both arms of the study (55% and 65%, respectively) are open to pharmacogenetic testing, and most (66% and 69%, respectively) believe that genetic testing has the potential to improve their medical care. Our results further support the potential for *CYP2D6* PGx testing to inform chronic pain medication management for poor metabolizers (PMs) and ultrarapid metabolizers (UMs). Future efforts to implement PGx testing in chronic pain management, however, must address patient concerns about genetic test result access and genetic discrimination.

## 1. Introduction

The Camden Opioid Research Initiative (CORI) is a multi-armed research study [1] consisting of a chronic pain cohort (Optimizing Pain Treatment in New Jersey (OPTIN)), an opioid use disorder (OUD) cohort (Genomics of Opioid Addiction Longitudinal Study (GOALS)), and a biobank resource of biospecimens collected from individuals who have died from an opioid overdose (CORI Biobank). As part of this initiative, we investigated the potential utility of pharmacogenetics (PGx) to inform opioid medication management in two clinical settings in the city of Camden: chronic pain management and opioid use disorder management. 

In theory, pharmacogenetics has the potential to improve medication management by increasing medication efficacy and decreasing adverse drug reactions [2,3,4,5,6,7,8,9]. There are, however, several important considerations for the translation of pharmacogenetics into clinical care [10], including whether there is clear, clinically interpretable evidence for a PGx role in medication management that is potentially useful in the relevant clinical care setting, and whether the relevant patients are willing to consent to PGx testing as part of their clinical care.

There is strong evidence that *CYP2D6* plays an important role in the metabolism of several commonly prescribed opioid pain medications, including oxycodone [11,12,13,14], codeine [15], tramadol [11,14,16], and hydrocodone [14,17,18]. The product of the *CYP2D6* gene, CYP2D6, is the primary enzyme that metabolizes each of these medications into the active metabolites that provide the majority of analgesia [14]. Specifically, CYP2D6 metabolizes oxycodone into oxymorphone [11], codeine into morphine [15], tramadol into O-desmethyltramadol [11], and hydrocodone into hydromorphone [18]. 

The Clinical Pharmacogenetics Implementation Consortium (CPIC) supports the implementation of PGx testing in clinical care. CPIC guidelines for *CYP2D6* and opioids [15] categorize four PGx phenotypes: ultrarapid metabolizer (UM), normal metabolizer (NM), intermediate metabolizer (IM), and poor metabolizer (PM) [15]; each of these phenotypes is defined by an enzyme activity score range (>2.25, 1.25 ≤ X ≤ 2.25, 0 < X <1.25, and 0, respectively) [15] and each activity score is calculated from both individual phased haplotypes, which are also referred to with diplotype *allele nomenclature [15]. CPIC further defines specific clinical guidelines for *CYP2D6* and codeine, tramadol, and hydrocodone [15], although as of the most recent opioid therapy guidelines, there is insufficient evidence for CPIC to provide clinical recommendations for oxycodone and *CYP2D6* [15]. 

One recent study in Florida assessed the potential clinical utility of *CYP2D6* pharmacogenetic testing in chronic pain management [14]. Smith et al. [14] compared pain intensity at baseline and at three months post-baseline between *CYP2D6*-guided treatment and usual care patients with chronic pain. Importantly, the authors found a significant pain reduction in *CYP2D6*-guided care patients (n = 51) relative to usual care patients (n = 19) in the subset that was IM and PM that was prescribed hydrocodone, tramadol, or codeine at baseline. 

With respect to OUD treatment, the Center for Healing at Cooper University Health Care routinely treats with buprenorphine. Buprenorphine is a partial, high-affinity mu-receptor agonist shown to be an effective treatment for persons with opioid use disorder [19]. While there is some evidence that variation in the *OPRM1* gene (rs1799971) potentially reduces the response to buprenorphine [20,21], and one or two copies of the *CYP3A4* variant rs2740574 T allele (which has also been used to define the *1b haplotype) may increase the rate of buprenorphine metabolism and thereby increase the rate of withdrawal as well as decrease its overall efficacy [21,22], this evidence is not strong enough for CPIC to offer clinical guidelines for either drug-gene pair. 

Given the established role of CYP2D6 in the metabolism of many opioids used in pain management, the encouraging chronic pain management PGx results [14] and the suggestive evidence for the utility of PGx in OUD management with buprenorphine, we investigated both of the abovementioned considerations: patient willingness to consent to PGx testing and the potential for PGx information to support opioid management. Below we describe OPTIN and GOALS patient baseline perceptions of pharmacogenetic testing in chronic pain management and opioid use disorder treatment, respectively. We further describe below the baseline medication management trends in OPTIN relative to CYP2D6 metabolizer status and GOALS with respect to *OPRM1* and *CYP3A4* variation.

## 2. Materials and Methods

GOALS and OPTIN are prospective, observational studies that include the collection of self-reported demographic and perception survey data at enrollment. In addition, participants are asked to grant access to their electronic health records for up to five years prior to baseline enrollment and up to 12 months after baseline enrollment [1].

### 2.1. Study Populations

CORI study enrollment criteria consist of an age of at least 18 years, English language proficiency, written informed consent, willingness to provide a saliva sample for DNA analysis, and either in-treatment status for chronic pain at Cooper University Health Care clinic (OPTIN) or in-treatment status for opioid use disorder at the Center for Healing at Cooper University Health Care as defined by DSM-5 (GOALS) [1]. 

The study was conducted according to the guidelines of the Declaration of Helsinki. The study protocol was reviewed and approved by Western IRB (IRB study # 1251380 for GOALS and # 20190132 for OPTIN), as well as by the IRBs of Cooper University Health Care (IRB study # 19-025 for GOALS and # 19-030 for OPTIN) and Coriell Institute for Medical Research (Study #R164 for GOALS and #R165 for OPTIN). Written informed consent was obtained from all subjects involved in the study after an in person review with study staff and after all questions were answered. Participants could choose not to participate in the study without any penalty or loss of benefits. In addition, participants could choose to withdraw from the study at any time without any penalty or loss of benefits.

### 2.2. Genetic and Non-Genetic Data Collection

In total, 119 participants enrolled in OPTIN, and 125 participants enrolled in GOALS. All of the GOALS and OPTIN participants answered demographic questions and completed a baseline genetic perception survey (see Section 3 for survey questions). Baseline medication prescription information was extracted from participants’ electronic health records. Participants additionally donated saliva for DNA extraction and analysis. Saliva was collected using Oragene-DNA saliva collection kits (DNA Genotek Inc., Ottawa, ON, Canada). DNA was extracted using the saliva kit reagents run on a Promega Maxwell magnetic bead automated DNA extraction instrument (Promega Coorporation, Madison, WI, USA). DNA quality and quantity were assessed with a Thermo Fisher Scientific NanoDrop One Microvolume UV-Vis Spectrophotometer (Thermo Fisher Scientific, Waltham, MA, USA) and an Invitrogen Qubit 4 Fluorometer using the Qubit dsDNA BR Assay Kit (Invitrogen, Waltham, MA, USA). The DNA was sent to Psomagen, Inc. for low pass (4x) whole genome sequencing (WGS); typically, WGS data are downsampled to ~16 GB, which is consistent with ~4x coverage. WGS data were processed for variant calling by Gencove (Gencove Inc., Long Island City, NY, USA), and pharmacogenetic variants were extracted with a custom bcftools [23] script for *CYP2D6*, *OPRM1* and *CYP3A4*. 

Of the 125 GOALS and 119 OPTIN participants, 118 GOALS and 114 OPTIN successful DNA extractions were sent for low pass sequencing, and the subset of 113 GOALS and 109 OPTIN WGS data passed Gencove quality checks for minimum coverage of total bases sequenced and variants covered with at least one read. Given the the 4x coverage data, we were unable to infer the *CYP2D6* copy number from the WGS data. *CYP2D6* copy number was assessed with an additional TaqMan^®^ Copy Number Assay from Thermo Fisher Scientific (assay id Hs00010001_cn) run on a QuantStudio ^TM^ Flex 6 Real-Time PCR system (Thermo Fisher Scientific, MA, USA), and copy number was calculated using CopyCaller ^®^ Software (Thermo Fisher Scientific, MA, USA).

All genetic data collection included a sample (n = 197) of DNAs previously collected and assayed for *CYP2D6* genetic variation and corresponding CYP2D6 metabolizer status in a previous study [5] to assess the genetic data quality of the 4x coverage variant calling against the set previously genotyped by the Affymetrix SNP 6.0 array. Mean SNP concordance was 98.95% across the overlapping SNPs (n = 762,863; C > G and A > T were excluded from this SNP quality assessment), and copy number concordance was 100%. Table S1 details how each metabolizer status was defined. 

All genetic data collected for this study were for research purposes only. No clinical PGx testing was performed, and no clinical PGx results were returned to clinicians or patients. All study results presented below consist of data collected at baseline.

### 2.3. Data Analysis

In total, participant baseline perception data from 119 participants enrolled in OPTIN and 125 participants enrolled in GOALS were retained for analysis. In total, genetic data from 113 OPTIN participants passed all of the quality control checks listed above and were retained for baseline medication management analysis. In total, genetic data from 102 GOALS participants being treated with sublingual buprenorphine passed all of the quality control checks listed above and were retained for baseline medication management analysis. All data analysis was performed using standard R functions (Version 3.6.3) [24], including the “boxplot” function, the “anova” function and the “lm” function.

## 3. Results

In total, data from 244 participants are included in the current study: 125 participants enrolled in GOALS, and 119 participants enrolled in OPTIN. As displayed in Table 1, the majority of patients in both arms of the study were female (56% and 63%, respectively), white (69% and 66%, respectively) and not Hispanic or Latino (74% and 82%, respectively). The mean age of GOALS participants was 41 years, and the mean age of OPTIN participants was 56 years. Below, we present a summary of patient pharmacogenetic perceptions within each independent clinical setting. We additionally present pharmacogenetic medication management trends within the chronic pain OPTIN cohort and the opioid use disorder GOALS cohort.

**Figure 1:**
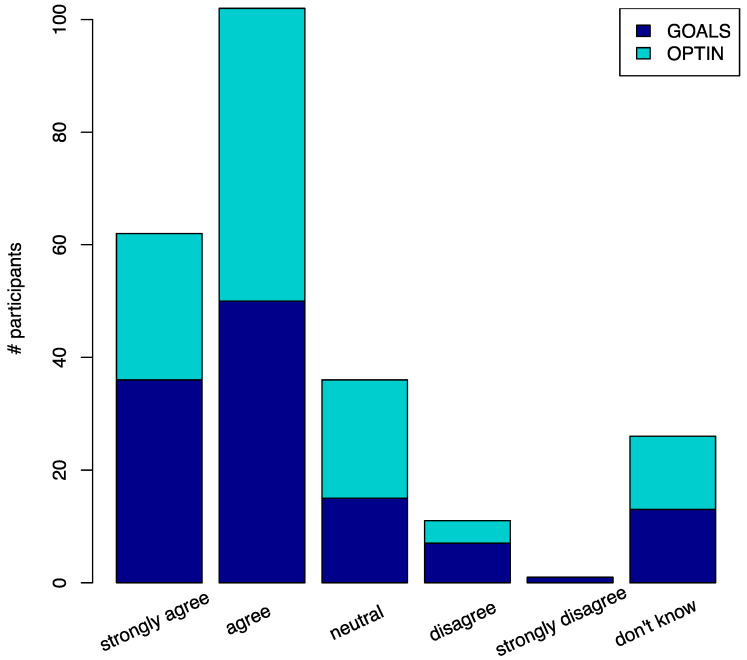
Visualization of participant response to “Genetics have the potential to improve my medical care”. The X-axis displays the range of potential statement responses, and the Y-axis displays the number of participants that chose a given response. GOALS participants are shaded in dark blue and OPTIN participants are shaded in aqua.

Table 2 summarizes the data collected through the participant baseline genetic perception survey. Each question is listed as a section header, each of the possible answers is listed below, and the corresponding counts and proportions of answers for each cohort are listed in each respective column. Most patients in OPTIN and GOALS (55% and 66%, respectively) are open to pharmacogenetic testing. Figure 1 visualizes the perception survey results for the question “Genetics have the potential to improve my medical care” with 67% of participants agreeing or strongly agreeing with this statement. No demographics were significantly associated (linear regression beta coefficient *p*-values > 0.05) with the answer to this survey question (age, self-reported gender, self-reported race, self-reported ethnicity, cohort). One patient strongly disagreed with this statement and reported concerns about genetic discrimination and about who would have access to their genetic test results. Out of 12 people that disagreed with the statement, five (45%) were concerned about who would have access to their genetic test results, and one (9%) was concerned about genetic discrimination.

**Figure 2:**
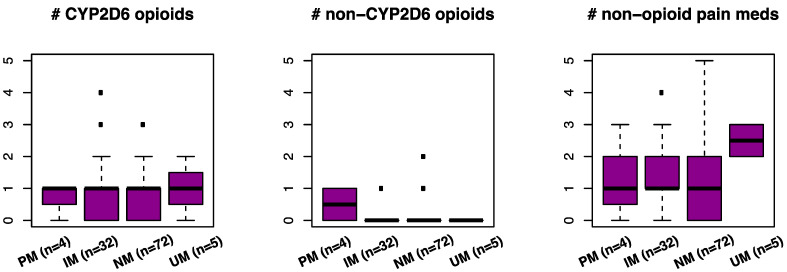
Visualization of the number of medications prescribed at baseline. The left panel displays the number of prescribed baseline opioid pain medications that require CYP2D6 for metabolism, the middle panel displays the number of prescribed baseline opioid pain medications that do not require CYP2D6 for metabolism, and the right panel displays the number of prescribed non-opioid pain medications. PM denotes poor metabolizer, IM denotes intermediate metabolizer, NM denotes normal metabolizer, and UM denotes ultra-rapid metabolizer. Each plotted box ranges from the 25th to the 75th percentile of the distribution. Whiskers extend in either direction to 1.5 times the interquartile range from each end of the box, or to the most extreme data point, whichever is less extreme.

Figure 2 visualizes the baseline medication trends for OPTIN participants in treatment for chronic pain broken down by CYP2D6 metabolizer status. Our OPTIN cohort only included four poor metabolizers (PM) and five ultra-rapid metabolizers (UM). We did not observe any statistically significant differences (ANOVA *p*-value > 0.05) between metabolizer status categories (PM, intermediate metabolizers or IMs, normal metabolizers or NMs, or UMs) for any of the medication categories (the number of prescribed baseline opioid pain medications that require CYP2D6 for metabolism, the number of prescribed baseline opioid pain medications that do not require CYP2D6 for metabolism, or the number of prescribed non-opioid pain medications); however, we note that according to the electronic health records, none of the four PMs were exclusively taking opioid pain medications that required CYP2D6 for metabolism at baseline. One of them was only being treated with non-opioid pain medications at baseline; one was being treated with a combination of opioid pain medication that requires CYP2D6 for metabolism as well as non-opioid pain medications at baseline; and the other two were being treated with a combination of opioid pain medication that requires CYP2D6 for metabolism, opioid pain medications that do not require CYP2D6 for metabolism, and non-opioid pain medications at baseline. We additionally note a visual trend for UMs to be treated with a larger number of non-opioid pain medications; however, this trend was not statistically significant (ANOVA *p*-value > 0.05).

CYP2D6 does not play a role in metabolizing buprenorphine, the primary medication used for opioid use disorder treatment at the Center for Healing at Cooper University Health Care; however, given the moderate CPIC cited evidence for variation in the *OPRM1* gene playing a role in buprenorphine [15,20,25], we explored the relationship between rs1799971 and baseline buprenorphine dosing (mg/day). For this comparison, we only included OUD patients receiving sublingual buprenorphine. We observed two of the potential three genotypes in our GOALS cohort (Figure S1) and found no difference in buprenorphine dosing between these two genotype categories (ANOVA *p*-value > 0.05). Finally, we explored the relationship between *CYP3A4* variation, particularly rs2740574, which defines the *1b allele [20], and baseline buprenorphine dosing (mg/day); we only included OUD patients receiving sublingual buprenorphine, observed all three genotypes in our GOALS cohort (Figure S2), and found no difference in buprenorphine dosing among these genotype categories (ANOVA *p*-value > 0.05). 

## 4. Discussion

While pharmacogenetics has the potential to improve medication management [2,3,4,5,6,7,8], the potential clinical utility should be considered within the context of each specific drug/gene/indication. Here, we investigated the potential clinical utility of pharmacogenetic information in opioid management for chronic pain and for the treatment of opioid use disorder.

We first considered evidence for a PGx role in medication management in each clinical setting. For chronic pain management with opioids that require CYP2D6 activity for analgesic benefit (PharmGKB evidence for hydrocodone, tramadol, as well as other published evidence for oxycodone [13] and tapentadol [26]), we anticipated that the identification of poor metabolizers that do not receive the analgesic benefits of these opioids could be useful in pain medications choice (i.e., clinicians could choose alternative opioids or non-opioid pain medications). We also asked whether the identification of ultra-rapid metabolizers could be useful in determining the dose and dose frequency as well as in pain medication choice (i.e., clinicians could choose alternative opioids or non-opioid pain medications if the analgesic effect was insufficient for a given patient). Our sample size of CYP2D6 PMs (N = 4) and UMs (N = 5) was too modest to identify any statistically significant differences in baseline medication management of opioids that require CYP2D6 for analgesic relief, opioids that do not require CYP2D6 for metabolism, or non-opioid pain medications; however, the observable trends (Figure 2) suggest that PMs are less likely to be exclusively treated with opioids that require CYP2D6 activity for pain relief. Indeed, none of the four PMs were exclusively taking opioid pain medications that required CYP2D6 at baseline, consistent with the findings of a prior proof of concept trial [14]. We also see a trend in UMs being treated with a larger number of non-opioid pain medications, which suggests that the length of analgesic relief from opioids may be a potential issue for these patients. We speculate that this subset of chronic pain patients could potentially benefit more from extended-release opioids if non-opioids are not working well.

We next assessed whether Cooper Hospital patients in treatment for chronic pain were willing to agree to PGx testing as part of their clinical care. Results from our OPTIN cohort survey indicate that the majority of them (66%) agree or strongly agree with the statement, “Genetics have the potential to improve my medical care”. This result is consistent with results from the Mayo Clinic RIGHT protocol (Right Drug, Right Dose, Right Time) study that found the majority of participants (87%) agreed with the statements regarding pharmacogenomic results having the potential to improve medication choice and dosing [27]. In response to the statement, “If there was a genetic test that would advise you and your doctor about your risk for developing opioid use disorder, how much would you want to get tested?”, a smaller majority (55%) chose extremely or considerably (Table 2). A non-trivial minority (13%) expressed concerns about genetic discrimination, and a larger minority (30%) expressed concerns about who would be able to access their genetic test results (Table 2). 

Published evidence for a potential PGx role in OUD medication management is less clear. Variation in the *OPRM1* gene (rs1799971) potentially reduces the response to buprenorphine [20,21], and the G allele associated with potentially reduced response has also been implicated as a risk allele for opioid use disorder in genome-wide association studies [28]. Similarly unclear is whether variation in the *CYP3A4* gene impacts buprenorphine response. One or two copies of the rs2740574 T allele (which has also been used to define the *1b haplotype) may increase the rate of buprenorphine metabolism and thereby increase the rate of withdrawal and decrease overall medication efficacy [21,22]. We did not find any significant differences or suggestive trends in baseline buprenorphine dosing in our modest cohort with respect to variation in either gene. We interpret these negative findings to result from some combination of our modest cohort size, non-genetic factors that influence buprenorphine treatment, and/or an absence of any meaningful role that variation at either gene plays in buprenorphine metabolism.

Perception survey results for the GOALS cohort (Cooper Hospital OUD patients) were similar to the OPTIN cohort. The majority of the GOALS cohort (69%) agree or strongly agree with the statement, “Genetics have the potential to improve my medical care”, and in response to “If there was a genetic test that would advise your doctor on which of the three opioid use disorder medication-assisted treatments (buprenorphine/Subutex, methadone/Dolophine or naltrexone/Vivitrol) would work best for you, how much would you want to get tested?”, a similar majority (66%) chose extremely or considerably (Table 2). A non-trivial minority (12%) expressed concerns about genetic discrimination, and a larger majority (21%) expressed concerns about who would be able to access their genetic test results (Table 2). Both concerns were similar to results in the OPTIN cohort described above.

A general patient PGx perception study conducted in Spain has shown a higher proportion (99%) of patients agreeing that pharmacogenetic testing is beneficial [29]. Studies of patient PGx perceptions in other specific clinical settings have similarly shown higher proportions of patients being open to PGx testing. For example, one study within the context of asthma treatment conducted in Germany showed that 96% of patients were open to PGx testing before receiving medication prescriptions [30]. Another literature review study of cancer patient perceptions found that a range of patients across studies (57–92%) believed that some form of personalized medicine testing was beneficial for treatment decision-making [31]. While these studies found a higher proportion of participants to be open to PGx testing relative to what we have found with the OPTIN and GOALS participants, there are some notable demographic and clinical differences. The studies with the highest proportion of open participants were conducted outside of the United States and importantly did not focus on opioid medication management. We believe the most likely explanation for the relatively smaller proportion of participants open to PGx testing in the current study is the concerns OPTIN and GOALS participants shared about access to genetic test results and genetic discrimination within the specific context of opioid medication management. 

To our knowledge, this is the first report of patient perceptions of pharmacogenetic testing in the context of chronic pain management and OUD treatment, and the majority of patients were open to PGx testing in both of these clinical settings. Patient concerns about genetic test result access and genetic discrimination, however, should be seriously considered and weighed against the potential benefits of clinical PGx testing in clinician discussions with their patients prior to clinical PGx testing. Concerns about PGx test result access and discrimination were previously documented in two general studies of pharmacogenetics [10,32].

Limitations of our study include the modest sample size recruited from a single clinical center, particularly the modest sample size of CYP2D6 poor metabolizers (N = 4) and ultra-rapid metabolizers (N = 5), which limit the statistical power we had to detect statistically significant differences in opioid and non-opioid pain medication management. In addition, we used cross-sectional, baseline electronic health record medication data, which did not include information about whether OPTIN and GOALS patients had reached stable dosing and treatment targets.

## 5. Conclusions

Our study of chronic pain and opioid use disorder patients treated at Cooper University Health Care in Camden City, NJ demonstrates that most patients in both arms of the study are open to pharmacogenetic testing and believe that genetic testing has the potential to improve their medical care. Our results further support the potential for *CYP2D6* PGx testing to inform chronic pain medication management for PMs and UMs. To realize the potential benefits, however, future efforts to implement *CYP2D6* PGx testing in chronic pain management must address patient concerns about genetic test result access and genetic discrimination. 

## Figures and Tables

**Table 1:** Participant Demographics.

	GOALS (N = 125)	OPTIN (N = 119)
Female	70 (56%)	75 (63%)
Male	55 (44%)	44 (37%)
Hispanic or Latino	28 (22%)	16 (13%)
Not Hispanic or Latino	93 (74%)	98 (82%)
Unknown	4 (3%)	5 (4%)
Asian	2 (2%)	1 (<1%)
Black or African-American	24 (19%)	27 (23%)
Native Hawaiian or other Pacific Islander	1 (<1%)	0 (0%)
Other	3 (2%)	2 (2%)
White(Caucasian)	86 (69%)	79 (66%)
More than one race	6 (5%)	7 (6%)
Unknown	3 (2%)	3 (3%)
Mean age at survey (years) ± SD	40.5 ± 11	55.8 ± 11

**Table 2:** Participant Baseline Genetic Perception Survey Results Summary.

	GOALS (N = 125)	OPTIN (N = 119)
“Have you been exposed to genetics before enrolling in the study?”		
Yes	36 (29%)	42 (35%)
No	80 (64%)	70 (59%)
Don’t know	7 (6%)	4 (3%)
No Answer	2 (2%)	3 (3%)
“Through which sources [have you been exposed to genetics before enrolling in the study]?” Please note, N is based on respondents who answered “Yes” to having been exposed to genetics before enrolling in the study (N = 36 for GOALS; N = 42 for OPTIN; participants could choose more than one response or could choose “other” and enter free text).		
Books	5 (14%)	5 (12%)
Genetic or personalized medicine websites	6 (17%)	4 (10%)
High school or college-level courses	22 (61%)	25 (61%)
Internet	9 (25%)	11 (27%)
News or magazine articles	9 (25%)	5 (12%)
Other: common knowledge	1 (3%)	0 (0%)
Other: jail	1 (3%)	0 (0%)
Other: previous genetic testing	5 (14%)	5 (12%)
Other: tv/documentaries	0 (0%)	2 (5%)
Other: direct to consumer genetic testing	0 (0%)	3 (7%)
Other: other study	0 (0%)	2 (5%)
Other: family	0 (0%)	1 (2%)
Other: previous clinical care	0 (0%)	0 (0%)
OPTIN: “If there was a genetic test that would advise you and your doctor about your risk for developing opioid use disorder, how much would you want to get tested?” GOALS: “If there was a genetic test that would advise your doctor on which of the three opioid use disorder medication-assisted treatments (buprenorphine/Subutex, methadone/Dolophine or naltrexone/Vivitrol) would work best for you, how much would you want to get tested?”		
Extremely	54 (43%)	33 (28%)
Considerably	28 (22%)	32 (27%)
Moderately	22 (18%)	20 (17%)
Slightly	7 (6%)	7 (6%)
Not at All	7 (6%)	13 (11%)
Don’t Know	5 (4%)	8 (7%)
Don’t Want to Answer	0 (0%)	2 (2%)
No Answer	2 (2%)	4 (3%)
“Compared to most people, how would you rate your knowledge of genetics?”		
Better than most people	13 (10%)	19 (16%)
About average	66 (53%)	63 (53%)
Less than most people	32 (26%)	26 (22%)
Don’t know	12 (10%)	7 (6%)
Don’t want to answer	0 (0%)	1 (1%)
No Answer	2 (2%)	3 (3%)
“Do you look for information about opioid use disorder?”		
Yes	84 (67%)	42 (35%)
No	37 (30%)	72 (61%)
Don’t know	2 (2%)	2 (2%)
No Answer	2 (2%)	3 (3%)
“Genetics have the potential to improve my medical care.”		
Strongly agree	36 (29%)	26 (22%)
Agree	50 (40%)	52 (44%)
Neutral	15 (12%)	21 (18%)
Disagree	7 (6%)	4 (3%)
Strongly disagree	1 (1%)	0 (0%)
Don’t know	13 (10%)	13 (11%)
Don’t want to answer	1 (1%)	0 (0%)
No answer	2 (2%)	3 (3%)
“I am concerned about who will be able to access my genetic test results.”		
Strongly agree	7 (6%)	9 (8%)
Agree	19 (15%)	27 (23%)
Neutral	19 (15%)	26 (22%)
Disagree	52 (42%)	37 (31%)
Strongly disagree	19 (15%)	11 (9%)
Don’t know	7 (6%)	6 (5%)
No answer	2 (2%)	3 (3%)
“Genetic testing puts people at risk for genetic discrimination.”		
Strongly agree	6 (5%)	2 (2%)
Agree	9 (7%)	13 (11%)
Neutral	20 (16%)	21 (18%)
Disagree	57 (46%)	46 (39%)
Strongly disagree	20 (16%)	13 (11%)
Don’t know	11 (9%)	20 (17%)
Don’t want to answer	0 (0%)	1 (1%)
No answer	2 (2%)	3 (3%)
“I would change my lifestyle based on genetic test results.”		
Strongly agree	10 (8%)	14 (12%)
Agree	37 (30%)	34 (29%)
Neutral	37 (30%)	26 (22%)
Disagree	19 (15%)	21 (18%)
Strongly disagree	6 (5%)	6 (5%)
Don’t know	13 (10%)	15 (13%)
Don’t want to answer	1 (1 %)	0 (0%)
No answer	2 (2%)	3 (3%)
“Were you ever told that you had a risk for developing opioid or substance use problems based on your family history?”		
Yes	39 (31%)	14 (12%)
No	78 (62%)	100 (84%)
Don’t know	6 (5%)	2 (2%)
No answer	2 (2%)	3 (3%)

## Data Availability

Information supporting reported results can be found in Table S1.

## Supplementary Materials

The following supporting information can be downloaded at: https://www.mdpi.com/article/10.3390/pharmaceutics14091863/s1. Table S1 includes details on how each PGx metabolizer status was defined in the current study. Figure S1 displays a boxplot visualization of baseline buprenorphine dosing across *OPRM1* rs1799971 genotype, and Figure S2 displays a boxplot visualization of baseline buprenorphine dosing across *CYP3A4* rs2740574 genotype.
