# Supplementary material for: Patient Perceptions and Potential Utility of Pharmacogenetic Testing in Chronic Pain Management and Opioid Use Disorder in the Camden Opioid Research Initiative
Source: Pharmaceutics. 2022 Sep 3;14(9):1863. doi: 10.3390/pharmaceutics14091863 (PMC9505214; doi:10.3390/pharmaceutics14091863)
Supplement: Supplementary file 1 [file pharmaceutics-14-01863-s001.zip › Kusic_et_al_Sup_figure_legends.pdf]

Figure S1. **Boxplot visualization of baseline buprenorphine dosing across *OPRM1* rs1799971 genotype.** The GOALS cohort included two genotypes (A/A on the left) and (A/G on the right). Each plotted box ranges from the 25th to the 75th percentile of the distribution. Whiskers extend in either direction to 1.5 times the interquartile range from each end of the box, or to the most extreme data point, whichever is less extreme.

Figure S2. **Boxplot visualization of baseline buprenorphine dosing across *CYP3A4* rs2740574 genotype.** The GOALS cohort included all three genotypes (C/C on the left), C/T in the middle and (T/T on the right). Each plotted box ranges from the 25th to the 75th percentile of the distribution. Whiskers extend in either direction to 1.5 times the interquartile range from each end of the box, or to the most extreme data point, whichever is less extreme.
