# Supplementary material for: Patient Perceptions and Potential Utility of Pharmacogenetic Testing in Chronic Pain Management and Opioid Use Disorder in the Camden Opioid Research Initiative
Source: Pharmaceutics. 2022 Sep 3;14(9):1863. doi: 10.3390/pharmaceutics14091863 (PMC9505214; doi:10.3390/pharmaceutics14091863)
Supplement: Supplementary file 1 [file pharmaceutics-14-01863-s001.zip › Kusic_et_al_TableS1.pdf]

Table S1. *allele defining variants

dbSNP ID	CYP2D6 Haplotype	Variant Type	Alleles	Functional Consequence	Activity Score
N/A	*1	N/A	N/A	N/A	1
rs35742686	*3	Deletion	CT>T	Frameshift Variant	0
rs3892097	*4	SNV	C>T	Splice Variant	0
rs5030655	*6	Deletion	CA>C	Frameshift Variant	0
rs5030656	*9	Deletion	CTTCT>CT	In-frame Deletion	0.5
rs1065852	*10	SNV	G>A / G>C	Missense Variant	0.25
rs16947	*17	SNV	G>A / G>T	Missense Variant	0.5
rs28371706	*17	SNV	G>A / G>C / G>T	Missense Variant	0.5
rs1058164	*29	SNV	G>C	Synonymous Variant	0.5
rs1135840	*29	SNV	C>G / C>T	Missense Variant	0.5
rs16947	*29	SNV	G>A / G>T	Missense Variant	0.5
rs59421388	*29	SNV	C>T	Missense Variant	0.5
rs61736512	*29	SNV	C>T	Missense Variant	0.5
rs28371725	*41	SNV	C>T	Intron Variant	0.5

Note that for *alleles with more than one defining SNP, the non-reference allele must be present for all defining variants and the listed activity score is for the entire haplotype. SNV stands for single nucleotide variant. Activity scores for each *allele were summed over both haplotypes for each participant to produce the total activity score per participant. Ultrarapid metabolizer (UM), normal metabolizer (NM), intermediate metabolizer (IM), and poor metabolizer (PM) were defined by the following combined activity score range, respectively: >2.25, 1.25 <= X <=2.25, 0 <= X <=1.25, and 0.
