# Supplementary figures and images for: Patient Perceptions and Potential Utility of Pharmacogenetic Testing in Chronic Pain Management and Opioid Use Disorder in the Camden Opioid Research Initiative
Source: Pharmaceutics. 2022 Sep 3;14(9):1863. doi: 10.3390/pharmaceutics14091863 (PMC9505214; doi:10.3390/pharmaceutics14091863)

# OPRM1 rs1799971 genotype

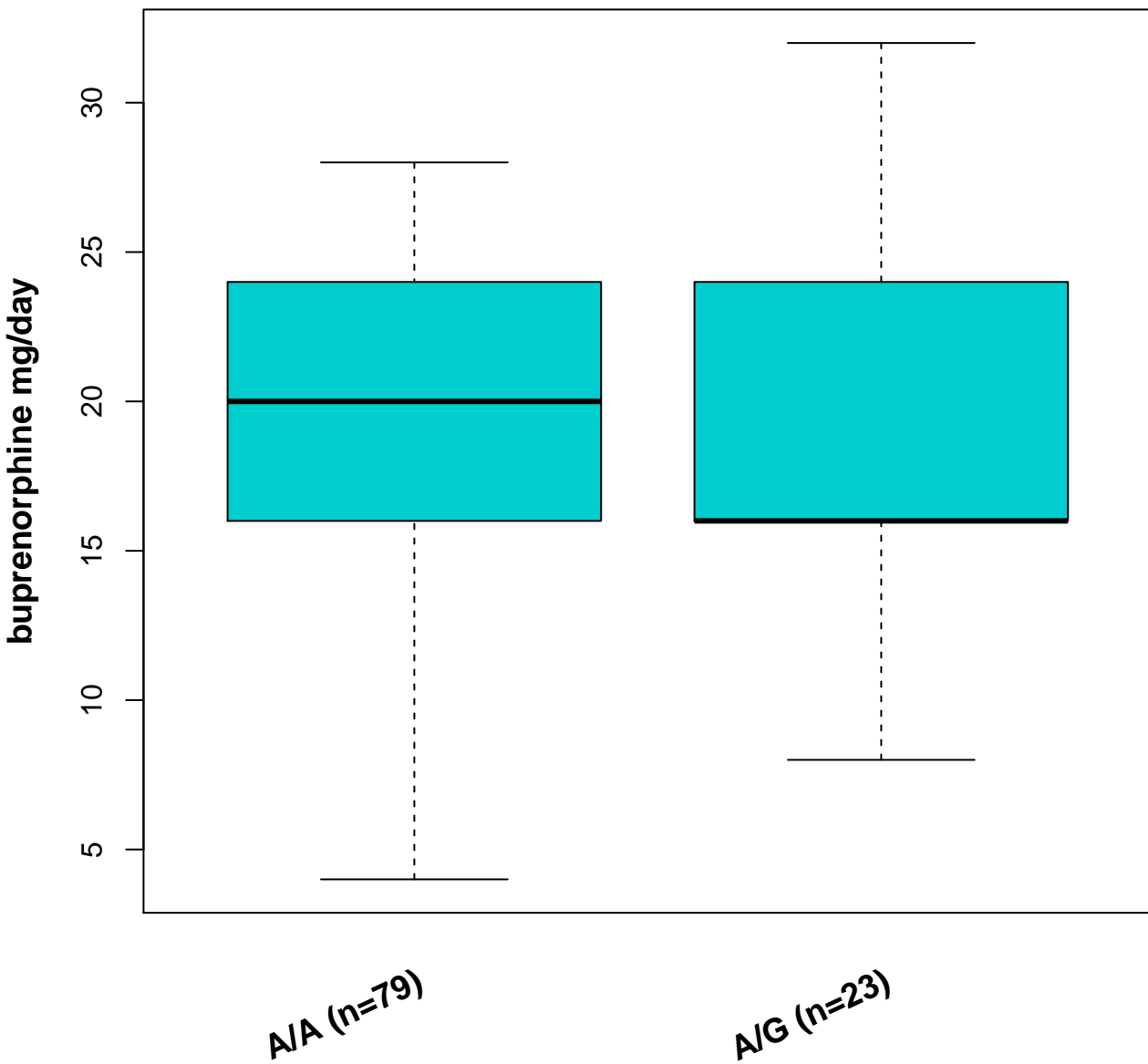

Supplement: Supplementary file 1 [file pharmaceutics-14-01863-s001.zip › Kusic_et_al_FigureS1.pdf]

# CYP3A4 rs2740574 genotype

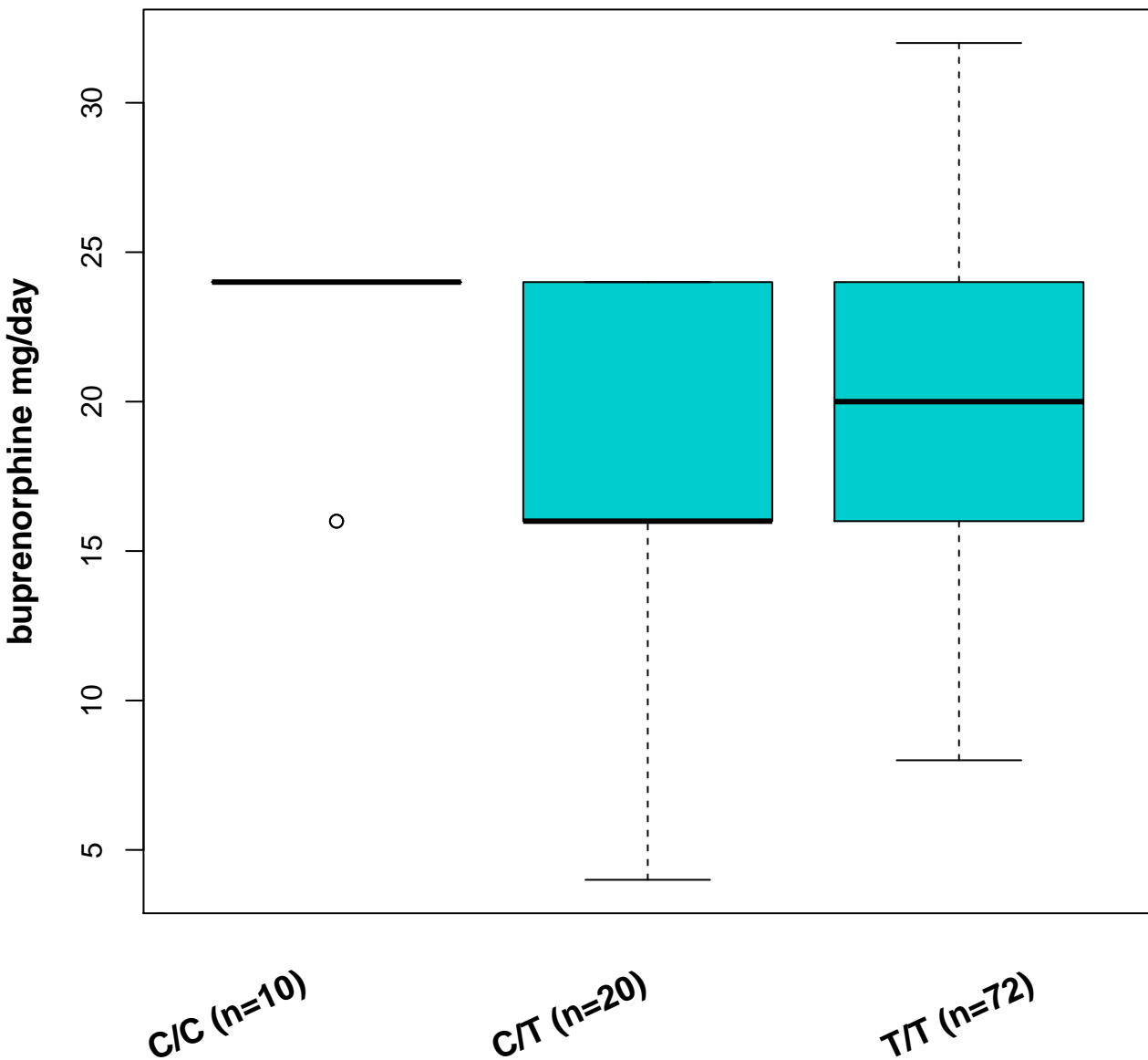

Supplement: Supplementary file 1 [file pharmaceutics-14-01863-s001.zip › Kusic_et_al_FigureS2.pdf]
